# Autism Spectrum Disorders and Malocclusions: Systematic Review and Meta-Analyses

**DOI:** 10.3390/jcm11102727

**Published:** 2022-05-11

**Authors:** Aline Barros, Paulo Mascarenhas, João Botelho, Vanessa Machado, Gabriela Balixa, Luísa Bandeira Lopes

**Affiliations:** 1 Centro de Investigação Interdisciplinar Egas Moniz, Egas Moniz—Cooperativa de Ensino Superior, CRL, 2829-511 Almada, Portugal; alineb.1410@gmail.com (A.B.); pmascarenhas@egasmoniz.edu.pt (P.M.); jbotelho@egasmoniz.edu.pt (J.B.); vmachado@egasmoniz.edu.pt (V.M.); 2 Evidence-Based Hub, Centro de Investigação Interdisciplinar Egas Moniz, Egas Moniz—Cooperativa de Ensino Superior, CRL, 2829-511 Almada, Portugal; gabrielabalixa@gmail.com

**Keywords:** autism spectrum disorder, malocclusion, oral health, systematic review

## Abstract

Studies heretofore have shown inconsistent results on the link of ASD to malocclusion. Herein, we aimed to compare the prevalence of malocclusion among children and adolescents with ASD compared with non-ASD healthy counterparts through a systematic review. The electronic search focused on five databases, PubMed, Web of Science, EMBASE, LILACS, and OpenGrey until January 2022, and followed the Preferred Reporting Items for Systematic Reviews and Meta-Analyses (PRISMA) guidelines (PROSPERO No. CRD42022298023). Observational and intervention studies that compared occlusion characteristics of ASD individuals under 18 years old with healthy controls were included. Pairwise random effects meta-analyses of odds ratio (OR) were performed. Methodological quality was assessed by using the Joanna Briggs Institute Critical Appraisal Checklist for cross-sectional studies. A total of thirteen studies were included for qualitative analysis, and seven for quantitative analysis. The results presented a great heterogeneity and moderate risk of bias; thus, it was not possible to state that there is a risk of malocclusion in individuals with ASD. Future studies should be carried out with strict criteria in the choice of samples, control group, and diagnosis of malocclusion in order to meet the necessary requirements for greater methodological quality.

## 1. Introduction

Autism Spectrum Disorder (ASD) is a group of multifactorial neurodevelopmental disorders that manifests early in lifetime and have significant lifelong impairments in social and professional contexts [1]. ASD is a new Diagnostic and Statistical Manual of Mental Disorders-5 (DSM-5) disorder that include autism, Asperger’s disorder, and pervasive developmental disorder not otherwise specified in the DSM-IV. It is characterized by deficits in two central domains: (1) deficits in social communication and social interaction and (2) repetitive and restricted patterns of behavior, interests, and activities [1].

The behavioral phenotype of ASD, regarding communication deficits, anxiety, fear, and dependence on parents and/or caregivers, often creates clinical challenges for health professionals [2]. On the oral health context, ASD patients have a greater tendency for parafunctional oral habits, such as bruxism, tongue thrust, or nonnutritive chewing, known to be indicators of malocclusion (such as anterior open bite, posterior crossbite, and excessive overjet) [3,4,5,6,7,8]. In addition, ASD as a neurodevelopmental disorder is also associated with a higher risk for speech problems [9].

The prevalence of malocclusion in ASD is a topic of high research interest yet with inconsistent evidence. While malocclusion has been shown to be significantly more probable to occur in ASD individuals than in healthy counterparts (including posterior crossbite, increased overjet, and severe maxillary crowding) [6,10], such a difference has also been reported as non-existent [8,11,12]. The nature of this association is not known, but literature points to the clinical management difficulties when treating ASD patients, which leads to a higher risk of undiagnosed clinical conditions. Regarding possible genetic alterations that may mediate this association, such information has never been explored. As such, and given the recent increase in number of published papers, this systematic review aimed to clarify whether malocclusion is more common in children and adolescents with ASD than in healthy controls.

## 2. Materials and Methods

### 2.1. Protocol and Registration

The protocol for this systematic review was defined by all authors and registered at the National Institute for Health Research PROSPERO, International Prospective Register of Systematic Review (http://www.crd.york.ac.uk/PROSPERO, ID Number: CRD42022298023). We prepared our review design on the Preferred Reporting Items for Systematic Reviews and Meta-Analysis (PRISMA) checklist [13].

### 2.2. Focused Question and Eligibility Criteria

We developed a protocol to answer the following PECO question: “Is there an increased risk of malocclusion in the autism spectrum patients?” The respective statements were as follows: individuals under 18 years of age (P, Population); diagnosis of ASD according to American Psychiatric Organization (1) (E, Exposure); healthy individuals (C, Comparison); prevalence of malocclusions (O, Outcome). The primary outcome was the class of malocclusion (type I, II, or III) in children and adolescents with ASD, where Class I or neutrocclusion takes into account that the mesiodistal relationship between the first molars is correct, that is, the mesiobuccal cusp of the maxillary first molar occludes in the direction of the mesiobuccal groove of the mandibular first molar. In turn, Class II or distoclusion is characterized by the distal position of the lower first molars in relation to the upper ones, unlike Class III or mesiocclusion, where the lower first molar is mesially related to the upper one. Secondary outcomes were alternative types of malocclusions, such as overbite, anterior crossbite, posterior crossbite, overcrowding, spacing, open bite, overjet, or diastema.

Studies were eligible for inclusion if they were: (1) published up until January 2022; (2) observational (cross-sectional, case-control) or intervention studies (randomized controlled trials (RCTs) or non-RCTs); (3) reporting at least one type of malocclusion; (4) demonstrating no history of orthodontic treatment. Studies with syndromic patients, case report studies, editorials and letters to the editor, reviews, and systematic reviews were excluded. This search was held without restrictions regarding language or year.

We have included both observational and intervention designs because restricting only to randomized studies of intervention would have provided a limited view of the summary on this matter, due to limited numbers of intervention trials and possibly unrepresentative estimations.

### 2.3. Search Strategy and Study Selection

The search and inclusion of studies was conducted by two independent reviewers (AB, LBL) in four different electronic platforms: PubMed via MEDLINE, Web of Science, and LILACS. Gray literature was searched using OpenGrey (http://www.opengrey.eu/ (accessed on 20 January 2022)). The search strategies were based on the following syntax: (“Autistic Disorder” OR “Autistic Spectrum Disorder) AND (malocclusion OR “open bite” OR crossbite OR “oral health”).

Two independent examiners performed in duplicate the assessment of titles and/or abstracts of retrieved studies independently (AB and LBL). For measurement reproducibility purposes, inter-examiner reliability following full-text assessment was calculated via kappa statistics. Any disagreements were resolved by discussion with a third author (VM).

### 2.4. Data Extraction Process and Data Items

Data extraction was performed by two reviewers independently and in duplicate (AB and LBL). Any paper classified as potentially eligible by either examiner was independently screened by the reviewers. All disagreements were resolved through discussion with a third reviewer (VM). The following information was gathered in general: description, research characteristics, methodology, and outcome measurements. The following standard information was extracted from each eligible study: first author’s name, country, year of publication, setting sampling, control group, subjects characteristics, Angle classification, overbite, overjet, crossbite, funding, and study outcomes.

### 2.5. Risk of Bias (RoB) Assessment

The methodological quality of the included observational and cross-sectional studies was appraised using the Joanna Briggs Institute (JBI) Critical Appraisal Checklist [14]. This tool was adapted from previously published systematic reviews. The items on the checklist were as follows: (1) clearly mention aim and justification of sample size; (2) sample randomization; (3) blind treatment allocation; (4) possibility of comparison between control and treatment groups; (5) baseline equivalence of control and treatment groups; (6) clearly describe the preparation protocol; (7) clearly report the experimental protocol; (8) measurement method and adequate statistical analysis. Each item was scored using a 2-point scale: 0—not reported or reported inadequately, and 1—reported and adequate. Any disagreements between the examiners were resolved through discussion with a third author. Studies with 12 to 11 points were considered to be of high quality, studies with 7 to 10 were of medium quality, and studies with 0 to 6 points were of low quality. For analytical cross-sectional studies, the Risk-Of-Bias VISualization (ROBVIS) tool was used as a tool to analyze the risk of bias (https://www.riskofbias.info/welcome/robvis-visualization-tool (accessed on 20 January 2022)).

### 2.6. Summary Measures and Synthesis of Results

Categorical variables were described by frequency and percentage and continuous variables were reported using the mean ± standard deviation (SD) and range.

To describe the occlusal disharmonies of ASD children and adolescents compared to healthy ones, an odds ratio (OR) with 95% confidence intervals (CI) was used. The OR was pooled using a random-effects model in R version 3.4.1 (R Studio Team 2018), using the ‘readxl’ package and using pairwise random-effects meta-analysis [15]. Forest plots were used to graphically present the pooled ORs [16], and *p*-values lower than 0.05 were statistically significant. The chi-square (χ^2^) test calculated overall homogeneity [17]. To assess sources of heterogeneity, the I^2^ index and Cochrane’s Q statistic (*p* < 0.1) were used [17]. To explore potential sources of heterogeneity, we conducted a sub-group analysis according to methodological quality of the included studies. Publication bias was planned if the meta-analysis included at least 10 studies [18].

## 3. Results

### 3.1. Study Selection

The initial dataset search retrieved 437 articles. After removing duplicates (*n* = 213), 197 were excluded after revising the title and/or abstract. From the overall 27 entries included for full article review eligibility, two were performed in adult populations [19,20], eleven did not report any type of malocclusion [5,21,22,23,24,25,26,27,28,29,30], and two had no ASD group of patients [9,31].

**Figure 1:**
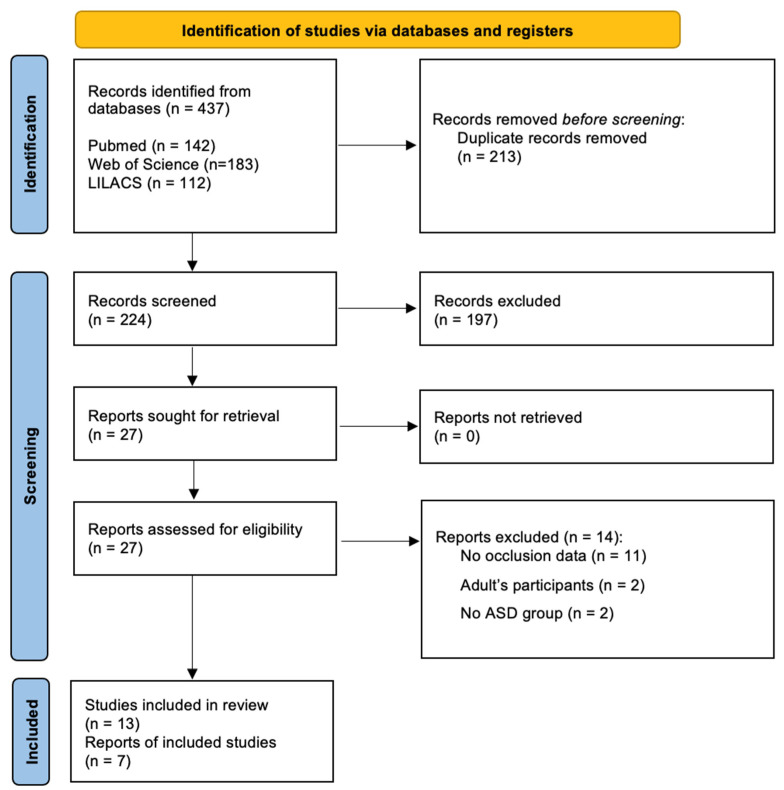
PRISMA flowchart depicting the workflow of the studies selection process based.

As a result, thirteen observational studies were included for qualitative synthesis, while 7 studies were included for quantitative estimations. The PRISMA diagram is shown in Figure 1. Inter-examiner reliability at the full-text screening was considered very substantial (kappa score = 0.915, 95% CI: 0.895–0.925).

### 3.2. Studies Characteristics

The characteristics of the included studies are presented in Table 1. The selected studies addressed the occlusion of individuals with ASD [19,32,33,34,35], and 8 were comparisons with non-ASD participants [3,4,6,7,8,11,12,36]. Of these, only Luppanapornlarp et al. [36] determined the occlusion according to the Dental Aesthetic Index (DAI) and, therefore, could not be included in the meta-analytic analysis.

Overall, from all 13 included studies, a total of 2390 participants were included in this SR, with 1622 ASD subjects (965 female and 345 males; 312 not reported sex) and 768 non-ASD participants (290 females and 184 males; 294 not reported sex). Four studies lacked sex information [3,12,32,33]. Among the ASD participants, the mean age ranged from 4.9 ± 0.8 years [11] to 12.8 ± 3.7 years [4] in ASD group, and from 8.4 ± 3.0 [7] to 12.8 ± 3.7 years [12] in non-ASD participants. Furthermore, studies were conducted in eleven countries across Europe, Asia, and the Americas. Notably, no study was performed in Oceania or Africa.

### 3.3. Methodological Quality of the Included Studies

**Figure 2:**
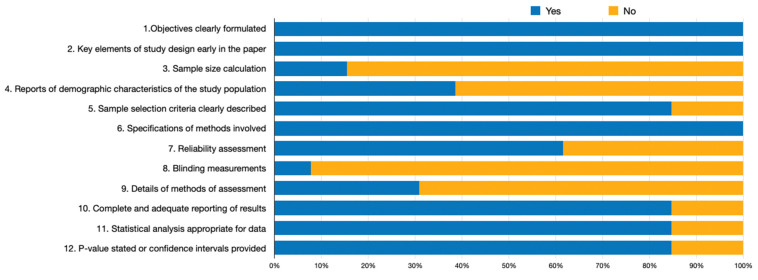
Assessment of the risk of bias in the included studies according to the percentage of the scores attributed to each evaluated study.

None of the included studies were classified as high quality, whereas twelve studies had moderate quality (2 scored with 10 points, 6 scored with 9 points, and 3 scored with 7 points) and two studies had low quality (scored with 5 points) (summarized in Figure 2 and detailed in the Supplementary Table S2). Good inter-examiner reliability was confirmed at the quality assessment (kappa score = 0.94, 95% CI: 0.84; 1.00).

All included studies showed clear objectives and key elements of study design (*n* = 13, 100%). The majority carefully described the sample selection criteria (*n* = 11, 84.6%), reported the results, and used a statistical appropriate analysis (*n* = 11, 84.6%). On the contrary, most articles failed on sample size justification (*n* = 11, 84.6%) and the demographic characteristics, namely the search period (*n* = 8, 61.5%), and only one study reported blindness during statistical analysis (*n* = 1, 7.7%) (12) (Figure 2 and Supplementary Table S2).

### 3.4. Outcomes Measures

#### 3.4.1. Malocclusion Class (Primary Outcome)

Angle’s classification was the most applied malocclusion classification; however, the results were inconsistent. Five studies compared the ASD Angle’s occlusion with non-ASD children, and we found no significant differences for class I (OR = 1.47, 95% CI: 0.47–4.59, *p* = 0.5101, I^2^ = 87.4%), class II (OR = 1.78, 95% CI: 0.97–3.24, *p* = 0.0619, I^2^ = 46.6%), or class III (OR = 0.87, 95% CI: 0.50–1.52, *p* = 0.0619, I^2^ = 0%) (Table 2). In all estimates, heterogeneity was considered high.

ASD patients were not associated with an increased risk of malocclusion, although only three studies (4,6,8) contributed to this statement (OR = 0.90; 95%CI: 0.24; 3.38; *p* = 0.8703) (Table 2, Supplementary Figure S1). Given that the number of included studies was below 10, publication bias was not deemed possible to carry out.

#### 3.4.2. Secondary Outcomes

Considering overjet, estimates depicted ASD children with a significantly higher risk for increased overjet (OR = 3.07, 95% CI: 1.10–8.57, *p* = 0.0043, I^2^ = 81.7%), and this was confirmed with a lower odds for normal overjet (OR = 0.28, 95% CI: 0.10–0.79, *p* = 0.0164, I^2^ = 72.9%) (Table 2).

The transverse dimension was explored through posterior crossbite. Subjects with ASD have not an increased risk of be diagnosis with buccal cusps of at least one of the maxillary posterior teeth (premolars and molars) occluded lingually or edge-to-edge to the buccal cusps of the mandibular teeth (OR = 1.38; 95% CI: 0.50–3.81, *p* = 0.5374, I^2^ = 39.2%) Table 2, Supplementary Figure S6). Additionally, for anterior cross bite, no significant differences were observed (OR = 1.72; 95% CI: 0.90–3.28, *p* = 0.1028, I^2^ = 34.4%). When observing both anterior and posterior, the same non-significance was reported (OR = 0.33; 95% CI: 0.11–1.00, *p* = 0.0508, I^2^ = 0.0%).

Regarding the vertical dimension, a vertical overlap of the maxillary central incisors over the mandibular central incisors when the posterior teeth were in the maximum intercuspation was considered. Estimates show that people with ASD do not have a significantly higher risk towards deep bite or increased overbite (OR = 1.19, 95% CI: 0.88–1.60, *p* = 0.2649, I^2^ = 0.0%), and open bite or decreased overbite (OR = 1.19, 95% CI: 0.58–2.43, *p* = 0.6413, I^2^ = 52.0%) (Table 2, Supplementary Figure S7).

Publication bias was not considered possible in the transversal and vertical dimensions and overjet analysis because the number of studies included was less than 10 in each analysis.

## 4. Discussion

### 4.1. Summary of Main Findings

The results of the present systematic review show that children and adolescents with ASD have an equal risk towards malocclusion compared with their non-ASD counterparts, except for increased overjet, where this risk was found to be significantly higher.

The scarcity of available studies, the low number of participants, the level of methodological heterogeneity, and the high variability limit the evidence certainty on the association between ASD and malocclusion, when compared to healthy controls. Despite these results, we anticipate that some part of these estimates may become significant with the increase of studies and participants, given the tendency of the estimates. Therefore, we strongly recommend caution when interpreting these results and the need for establishing preventive screening measures towards malocclusion in ASD. This means that oral care providers shall be more aware of potential orthodontic conditions in this particular group given their social communication and interaction limitations.

### 4.2. Implications for Practice and Research

As previously mentioned, ASD is a developmental disability that challenges its clinical care and management in the dental setting. This developmental impairment is often followed by inadequate oral hygiene habits as a result of the difficulties encountered by trainers and parents and, as a consequence, increases the risk for periodontal disease and dental caries [37,38]. Along with these conditions, malocclusion traits are very common conditions found in the general population, and the same is no exception in ASD; however, due to the difficulties already discussed, preventive screening is often hard to achieve. Furthermore, and considering the genetic cause of these syndromes, several studies have questioned whether ASD is associated to higher predisposition to malocclusion traits. Our results do not support such hypothesis but present preliminary estimates that may become significant with the progression of studies on this topic.

With all this in mind, it is our understanding that if the possibility is not completely excluded, and if some malocclusion profiles have higher predisposition, the most acceptable conduct should be to assume that those with ASD may have similar risks as healthy controls. Nevertheless, their social communication and interaction limitations that make the normal dental therapeutic setting difficult may increase the level of clinical priority towards preventive and early screening triage.

Despite all malocclusion traits included, retrognathia [9] and incisors inclination and teeth rotation [34] have only been reported once, without significant differences. However, this shall be expanded in the future, along with the remaining traits.

### 4.3. Strengths and Limitations

This study was conducted and reported following PRISMA, a strict and widely advised guideline that has increased the robustness and decreased reporting errors. Furthermore, a comprehensive literature search with a meticulous predefined protocol was conducted. Nevertheless, there are limitations to be discussed mostly related to the studies included. Most of them had a low number of ASD participants included, which may have limited the representativeness, and this is a point to be improved. Likewise, several studies did not employ the same classification of malocclusions and occlusal disharmonies. It is essential that there is a standardization of the applied classification, in order to present consistent results. Moreover, parafunctional habits were not evaluated and it may be of clinical importance to explore its confounding role in the association levels. The abovementioned shortcomings may have contributed to the observed heterogeneity.

Thus, future studies shall focus on data representativeness and method standardization to ensure more homogeneous evidence-based results in the future. This information is extremely relevant to clinicians and can assist in the development and implementation of future oral health programs tailored to the particularities and needs of ASD.

## 5. Conclusions

ASD presents a higher risk for increased overjet, but not for the remaining malocclusion types. Given the observed methodological heterogeneity, herein we provide instructions to standardize future studies on this topic.

## Figures and Tables

**Table 1:** Characteristics of the included studies.

Author, Year (Country)	Funding	Search Period	ASD/Non-ASD Participants (N Total [Male/Female])	Age Range (Years) (ASD/Non-ASD)	Occlusion ASD Participants (*n* [%])	Occlusion Non-ASD Participants (*n* [%])	Main Results
Bagattoni et al. 2021 (Italy) [7]	NR	January 2015 to March 2018	64 (42/22)/ 64 (37/27)	9.0 ± 2.9/8.4 ± 3.0	Class I—34 (70.0%); Class II—13 (26.0%); Class III—2 (4.0%); Posterior crossbite—9 (14.0%); **Overbite** Anterior open bite—12 (19.0%); Deep bite—9 (14.0%).	Class I—38 (76.0%); Class II—9 (18.0%); Class III—3 (6.0%); Posterior crossbite—10 (17.0%); **Overbite** Anterior open bite—3 (5.0%); Deep bite—10 (17.0%).	The difference between the two groups was not statistically significant in the overall analysis (*p* > 0.05), except on the anterior open bite (*p* = 0.013)
Farmani et al. 2020 (Iran) [8]	Vice Chancellery of Shiraz University of Medical Sciences, Shiraz, Iran (grant number: 16499).	June 2018 to October 2018	47 (36/11)/ 49 (28/27)	10.7 ± 2.1/9.5 ± 1.3	Malocclusion—35 (76.1%); Class I—20 (55.6%); Class II—16 (44.4%); Class III—7 (25.9%); **Overjet** Normal—20 (55.6); Increased—16 (44.4%); Decreased—7 (25.9%); **Overbite** Normal—24 (66.7%); Increased—12 (33.3%); Decreased—7 (22.6%); **Crossbite** Anterior and posterior—3 (6.4%)	Malocclusion—38 (79.2%); Class I—30 (88.2%); Class II—4 (11.8%); Class III—13 (30.2%); **Overjet** Normal—30 (88.2%); Increased—4 (11.8%); Decreased—13 (30.2%); **Overbite** Normal—24 (75.0%); Increased—8 (25.0%); Decreased—13 (35.1%); **Crossbite** Anterior and posterior—7 (14.3%)	Increased overjet and Class II molar relationship were the most prevalent malocclusions in the ASD group compared with control group (*p* = 0.03). ASD children were 6 times more likely to have increased overjet than those in the control group (OR: 6.0; 95% CI: 1.7–20.6). There was no statistically significant difference between the two groups in terms of crossbite and overbite.
Alkhabuli et al. 2019 (United Arab Emirates) [33]	None	NR	9 (NR/NR)/Not Present	NR/Not Present	Class II—38.0%; Class III—25.0%; Class II/III—38.0%	Not Present	Class II and Class III malocclusions among ASD patients are frequent
Kuter 2019 (Turkey) [12]	NR	NR	285 (NR/NR)/ 122 (NR/NR)	range 12–16 years	Open bite—16 (5.7%)	Open bite—6 (4.9%)	No significant difference in the proportion of open bite was identified (*p* > 0.05)
Leiva-García et al. 2019 (Spain) [4]	Mutua Madrileña Research Foundation.	January 2016 to December 2017	51 (37/13)/ 93 (50/43)	12.8 ± 3.7/12.8 ± 3.7	No malocclusion—12 (24.0%); Class I crowding—20 (40.0%); Class II—6 (10.0%); Class III—3 (6.0%); Open bite—9 (18.0%); Crossbite—1 (2.0%)	No malocclusion—46 (49.5%); Class I crowding—8 (8.6%); Class II—12 (12.9%); Class III—8 (8.6%); Open bite—4 (4.3%); Crossbite −8 (8.6%)	Malocclusion and open bite were more prevalent in the ASD group than in the control group (*p* = 0.000).
Orellana et al. 2019 (Chile) [19]	Comisión Nacional de Investigación Científica y Tecnológica, Chile. Proyecto FONIS SA15I20110.	2016–2017	123 (102/21)/ Not Present	9.4 ± 4.3/Not Present	Deep/ogival palate—64 (52.0%); Anterior open bite—7 (5.7%); **Crossbite** Anterior—10 (8.1%); Posterior—4 (3.3%).	Not Present	A high percentage of deep/ogival palate was found in this population
Önol & Kurzioğlu 2018 (Turkey) [3]	None	March to July 2016.	33 (NR/NR)/ 33 (NR/NR)	10.5 ± 2.9/10.2 ± 2.5	Class I—23 (69.8%); Class II division I—6 (20.6%); Class II division II—2 (4.8%); Class III—2 (4.8%); Cross bite—0 (0.0%); Open bite—1(1.6%); Deep bite—0 (0.0%); High arch palate—2 (6.3%)	Class I—29 (86.5%); Class II division I—3 (8.1%); Class II division II—1 (3.6%); Class III—1 (1.8%); Cross bite—1 (3.0%); Open bite—1 (1.8%); Deep bite—2(5.4%); High arch palate—1(0.9%)	Crossbite and deep bite were more prevalent in the non-ASD group than in the ASD group (*p* = 0.013). No significant differences were found in Angle’s molar relationship.
Alkhadra 2017 (Saudi Arabia) [35]	None	NR	100 (65/35)/ Not Present	NR/Not Present	Crossbite—10 (10.0%) **Overjet** Normal—84 (84.0%); Increased—16 (16.0%); **Overbite** Normal—55 (55.0%); Increased—4 (4.0%); Permanent dentition Right/Left Class I—40/41 (40.0%/41.0%); Right/Left Class II—16/13 (16.0%/13.0%); Right/Left Class III—3/5 (3.0%/5.0%);	Not Present	ASD children exhibited more of class I malocclusion.
DeMattei et al. 2017 (United Arab Emirates) [32]	The Autism Project.	NR	39 (NR/NR)/ Not Present	NR/Not Present	Class I—18 (46.2%); Class II—14 (35.9%); Class III—7 (17.9%); Cross bite—5 (12.8%); Crowding—1 (2.6%)	Not Present	No significant difference in the oral health status of children with an ASD when comparing younger children to older children or when comparing children with an ASD who resided with their parents to those who lived at the residential school
Fontaine-Sylvestre et al. 2017 (Canada) [6]	NR	January 2013 to August 2015	99 (78/21)/ 101 (83/18)	11.0 ± 3.7/11.0 ± 3.8	Class I—37 (42.5%); Class II—37 (42.5%); Class III—13 (14.9%); Midline deviation (>4 mm)—35 (38.9%); Midline deviation (<4 mm)—55 (61.1%). **Crossbite** Anterior—8 (8.1%); Posterior—13 (13.1%); **Overbite** Anterior Open bite—8 (8.1%); Posterior Open bite—3 (3.0%); Normal—67 (77.0%); Increased (>65%)—12 (13.8%); Decreased (≤0%)—8 (9.2%); **Overjet** Normal—49 (54.4%); Increased (>4 mm)—35 (38.9%); Decreased (<1 mm)—6 (6.7%); **Crowding** Minimal Maxillary—29 (29.3%); Moderate Maxillary—4 (4.0%); Severe Maxillary—5 (5.1%); Minimal Mandibular—36 (36.4%); Moderate Mandibular—8 (8.1%); Severe Mandibular—4 (4.0%)	Class I—51 (56.0%); Class II—30 (33.0%); Class III—10 (11.0%); Midline deviation (<4 mm)—69 (68.3%); Midline deviation (>4 mm)—32 (31.7%). **Crossbite** Anterior—6 (5.9%); Posterior—5 (4.9%); **Overbite** Anterior open bite—6 (3.9%); Normal– 79 (79.0%); Increased (>65%)—15 (15.0%); Decreased (≤0%)—6 (6.0%); **Overjet** Normal—85 (85.1%); Increased (>4 mm)—11 (10.9%); Decreased (<1 mm)—4 (4.0%); **Crowding** Minimal Maxillary—20 (19.8%); Moderate Maxillary—17 (16.8%); Severe Maxillary—1 (1.0%); Minimal Mandibular—23 (22.8%); Moderate Mandibular—25 (14.9%); Severe Mandibular—3 (3.0%)	Midline deviation (33.5%) was the most common trait in this population. Children with ASD had a significantly higher prevalence of posterior crossbite (*p* = 0.03), increased overjet (*p* < 0.001), and severe maxillary crowding (*p* = 0.006)
Du et al. 2015 (Hong Kong) [11]	General Research Fund (17116014) of the Research Grant Council of Hong Kong.	NR	257 (217/40)/ 258 (218/40)	4.9 ± 0.8/NR	**Overbite** Deep bite—95 (37.0%); Anterior open bite—6 (2.3%); **Overjet** Increased—48 (18.7%); **Crossbite** Anterior—36 (14.0%); Posterior—0 (0.0%)	**Overbite** Deep bite—80 (31.1%); Anterior open bite—10 (3.9%); **Overjet** Increased Overjet—38 (14.8%); **Crossbite** Anterior—28 (10.90%); Posterior—1 (0.4%)	No statistically significant difference was found between the two groups (*p* > 0.05)
Rekha et al. 2012 (India) [34]	NR	NR	483 (363/120)/ Not Present	NR/Not Present	Primary dentition Crowding—0 (0.0%); Proinclination—3 (0.6%); Anterior open bite—0 (0.0%); Rotation—0 (0.0%); Mixed dentition Crowding—21 (4.34%); Proinclination—15 (3.1%); Anterior open bite—3 (0.6%); Rotation—3 (0.6%); Permanent dentition Crowding—51 (10.5%); Proinclination—42 (8.6%); Anterior open bite—0 (0.0%); Rotation—6 (1.2%)	Not Present	Children with permanent dentition had more malocclusion (71.15%)
Luppanapornlarp et al. 2010 (Tailand) [36]	NR	NR	32 (25/7)/ 48 (19/29)	9.7 ± 1.2/9.9 ± 1.1	DAI score ≤ 25—12 (37.5%); DAI score 26–30—8 (25.0%); DAI score 31–35—7 (22.0%); DAI ≥ 36—5 (15.5%)	DAI score ≤ 25—14 (29.0%); DAI score 26–30—14 (29.0%); DAI score 31–35—13 (27.0%); DAI ≥ 36—7 (15.0%)	In ASD children, malocclusion symptoms such as missing teeth, spacing, diastemas, reverse overjet, open bite, and Class II molar relationship tendency were found at a higher percentage than in the control group

ASD—Autistic Spectrum Disorder; DAI—Dental Aesthetic Index; *n*—number of participants; non-ASD—non-Autistic Spectrum Disorder; NR—Not Reported.

**Table 2:** Occlusion among children and adolescents with and without ASD.

Variable	N Studies	N of Participants (ASD/Controls)	OR	95% CI	*p*-Value	I^2^ (%)
Malocclusion	3	197/243	0.90	0.24; 3.38	0.8703	89.5
Class I	5	275/324	1.47	0.47; 4.59	0.5101	87.4
Class II	5	275/324	1.78	0.97; 3.24	0.0619	46.6
Class III	5	275/324	0.87	0.50; 1.52	0.6346	0.0
**Crossbite**						
Anterior Crossbite	3	420/423	1.72	0.90; 3.28	0.1028	34.4
Posterior Crossbite	3	405/409	1.38	0.50; 3.81	0.5374	39.2
Anterior + Posterior Crossbite	3	131/175	0.33	0.11; 1.00	0.0508	0.0
**Overbite**						
Deep bite or increased overbite	5	496; 501	1.19	0.88; 1.60	0.2649	0.0
Open bite or decreased overbite	6	768; 652	1.19	0.58; 2.43	0.6413	52.0
**Overjet**						
Increased overjet	3	399; 406	**3.07**	**1.10; 8.57**	**0.0043**	81.7
Normal overjet	2	142; 148	**0.28**	**0.10; 0.79**	**0.0164**	72.9
Decreased overjet	2	142; 148	0.83	0.28; 2.48	0.7388	43.4

ASD—Autistic Spectrum Disorder; CI—Confidence Interval; OR—Odds Ratio.

## Data Availability

Not applicable.

## Supplementary Materials

The following supporting information can be downloaded at: https://www.mdpi.com/article/10.3390/jcm11102727/s1. Table S1. List of potentially relevant studies not included in the systematic review, along with the reasons for exclusion. Table S2. Quality assessment of Selected Full Text Article. Figure S1. Forest plots for Odds Ratio of malocclusion of ASD versus control group. Figure S2. Forest plots for Odds Ratio of overjet of ASD versus control group. Subgroup analysis according to the increased, normal and decreased overjet. Figure S3. Forest plots for Odds Ratio of Class I of ASD versus control group. Figure S4. Forest plots for Odds Ratio of Class II of ASD versus control group. Figure S5. Forest plots for Odds Ratio of Class III of ASD versus control group. Figure S6. Forest plots for Odds Ratio of crossbite of ASD versus control group. Subgroup analysis according to the anterior, posterior, and anterior + posterior crossbite. Figure S7. Forest plots for Odds Ratio of overbite of ASD versus control group. Subgroup analysis according to the deep bite or increased overbite, and open bite or decreased overbite.
