# Supplementary material for: Autism Spectrum Disorders and Malocclusions: Systematic Review and Meta-Analyses
Source: J Clin Med. 2022 May 11;11(10):2727. doi: 10.3390/jcm11102727 (PMC9147636; doi:10.3390/jcm11102727)
Supplement: Supplementary file 1 [file jcm-11-02727-s001.zip › jcm-1700820-supplementary.pdf]

Online Supplemental Information

Summary

Table S1. List of potentially relevant studies not included in the systematic review, along with the reasons for exclusion.......... 2

Table S2. Quality assessment of Selected Full Text Article. 4

Figure S1. Forest plots for Odds Ratio of malocclusion of ASD versus control group....... 5

Figure S2. Forest plots for Odds Ratio of overjet of ASD versus control group. Subgroup analysis according to the increased, normal and decreased overjet. 5

Figure S3. Forest plots for Odds Ratio of Class I of ASD versus control group. 5

Figure S4. Forest plots for Odds Ratio of Class II of ASD versus control group. 6

Figure S5. Forest plots for Odds Ratio of Class III of ASD versus control group. 6

Figure S6. Forest plots for Odds Ratio of crossbite of ASD versus control group. Subgroup analysis according to the anterior, posterior, and anterior + posterior crossbite.......... 6

Figure S7. Forest plots for Odds Ratio of overbite of ASD versus control group. Subgroup analysis according to the deep bite or increased overbite, and open bite or decreased overbite.......... 7

Table S1. List of potentially relevant studies not included in the systematic review, along with the reasons for exclusion.

Year	Reference	Reason
2021	Kuter B, Uzel I. Evaluation of oral health status and oral disorders of children with autism spectrum disorders by gender. Arch Pediatr. 2021 Jan;28(1):33-38. doi: 10.1016/j.arcped.2020.10.009. Epub 2020 Nov 27. PMID: 33257211	No occlusion data
2020	Koskela A, Neittaanmäki A, Rönnberg K, Palotie A, Ripatti S, Palotie T. The relation of severe malocclusion to patients' mental and behavioral disorders, growth, and speech problems. Eur J Orthod. 2021 Apr 3;43(2):159-164. doi: 10.1093/ejo/cjaa028.	No ASD group
2020	Bossù M, Trottini M, Corridore D, Di Giorgio G, Sfasciotti GL, Palaia G, Ottolenghi L, Polimeni A, Carlo SD. (2020). Oral Health Status of Children with Autism in Central Italy. Applied Sciences. 10. 2247. 10.3390/app10072247	No occlusion data
2019	Junior RCM, Rangel ML, Carvalho LGA, Figueiredo SC, Ribeiro ILA, Castro RD. Social, Educational and Dental Profiles of Brazilian Patients with Special Needs Attended at a Center for Dental Specialties. Pesquisa Brasileira em Odontopediatria e Clínica Integrada [online]. 2019, v. 19, e3819. doi: 10.4034/PBOCI.2019.191.55.	No occlusion data
2019	Makkar A, Indushekar KR, Saraf BG, Sardana D, Sheoran N. A cross sectional study to evaluate the oral health status of children with intellectual disabilities in the National Capital Region of India (Delhi-NCR). J Intellect Disabil Res. 2019 Jan;63(1):31-39. doi: 10.1111/jir.12553.	No occlusion data
2019	Suhaib F, Saeed A, Gul H, Kaleem M. Oral assessment of children with autism spectrum disorder in Rawalpindi, Pakistan. Autism. 2019 Jan;23(1):81-86. doi: 10.1177/1362361317730299. Epub 2017 Oct 27. PMID: 29076744.	No occlusion data
2018	Naidoo M, Singh S. The Oral health status of children with autism Spectrum disorder in KwaZulu-Nata, South Africa. BMC Oral Health. 2018 Oct 12;18(1):165. doi: 10.1186/s12903-018-0632-1.	No occlusion data
2017	Nunes R, Simões PW, Pires PDS, Rosso MLP. Prevalência de alterações bucais em pessoas com deficiência na clínica da Universidade do Extremo Sul Catarinense. Revista de Odontologia da Universidade Cidade de São Paulo, [S.l.], v. 29, n. 2, p. 118 - 128, nov. 2017. ISSN 1983-5183.	No occlusion data
2015	Blomqvist My, Bejerot S, Dahllöf G. A cross-sectional study on oral health and dental care in intellectually able adults with autism spectrum disorder. BMC Oral Health. 2015 Jul 15;15:81. doi: 10.1186/s12903-015-0065-z.	Adult's participants
2014	Khatib AAE, Tekeya MME, Tantawi MAE, Omar T. Oral health status and behaviours of children with Autism Spectrum Disorder: a case-control study. Int J Paediatr Dent. 2014 Jul;24(4):314-23. doi: 10.1111/ipd.12067.	No occlusion data
2014	Vellappally S, Gardens SJ, Al Kheraif AA, Krishna M, Babu S, Hashem M, Jacob V, Anil S. The prevalence of malocclusion and its association with dental caries among 12-18-year-old disabled adolescents. BMC Oral Health. 2014 Oct 1;14:123. doi: 10.1186/1472-6831-14-123.	No ASD group
2012	Orellana L-M, Silvestre F-J, Martínez-Sanchis S, Martínez-Mihi V, Bautista D. Oral manifestations in a group of adults with autism spectrum disorder. Med Oral Patol Oral Cir Bucal. 2012 May 1;17(3):e415-9. doi: 10.4317/medoral.17573.	Adult's participants
2011	Jaber MA, Sayyab M, Abu Fanas SH. Oral health status and dental needs of autistic children and young adults. J Investig Clin Dent. 2011 Feb;2(1):57-62. doi: 10.1111/j.2041-1626.2010.00030.x.	No occlusion data

2011	Jaber MA. Dental caries experience, oral health status and treatment needs of dental patients with autism. J Appl Oral Sci. 2011 May-Jun;19(3):212-7. doi: 10.1590/s1678-77572011000300006.	No occlusion data
2001	Fahlvik-Planefeldt C, Herrström P. Dental care of autistic children within the non-specialized Public Dental Service. Swed Dent J. 2001;25(3):113-8.	No occlusion data

Table S2. Quality assessment of Selected Full Text Article.

Author et al., Year	Item 1	Item 2	Item 3	Item 4	Item 5	Item 6	Item 7	Item 8	Item 9	Item 10	Item 11	Item 12	Total	Quality assessment
Bagattoni et al. 2021	1	1	1	1	1	1	1	0	0	1	1	1	10	Medium quality
Farmani et al. 2020	1	1	0	1	1	1	1	0	1	1	1	1	10	Medium quality
Kuter 2019	1	1	0	0	0	1	0	1	0	0	1	1	7	Medium quality
Leiva-García et al. 2019	1	1	0	1	1	1	1	0	0	1	1	1	9	Medium quality
Önol & Kurzioğlu 2018	1	1	0	0	1	1	0	0	0	1	1	1	7	Medium quality
Fontaine-Sylvestre et al. 2017	1	1	0	1	1	1	0	0	1	1	1	1	9	Medium quality
Du et al. 2015	1	1	1	0	0	1	1	0	0	1	1	1	9	Medium quality
Alkhabuli et al. 2019	1	1	0	0	1	1	1	0	0	0	0	0	5	Low quality
Alkhadra 2017	1	1	0	0	1	1	0	0	0	1	0	0	5	Low quality
DeMattei et al. 2007	1	1	0	0	1	1	1	0	1	1	1	1	9	Medium quality
Luppanapornlarp et al. 2010	1	1	0	0	1	1	1	0	1	1	1	1	9	Medium quality
Orellana et al. 2019	1	1	0	1	1	1	1	0	0	1	1	1	9	Medium quality
Rekha et al. 2012	1	1	0	0	1	1	0	0	0	1	1	1	7	Medium quality

Item1. Are the objectives clearly formulated?; Item 2. Are there key elements of study design early in the paper?; Item 3. Was the sample size calculated?; Item 4. Does the study report demographic characteristics of the study population?; Item 5. Were the sample selection criteria clearly described?; Item 6. Does the study describe specifications of material and methods involved including how and when measurements were taken?; Item 7. Was there a reliability assessment, with adequate level of agreement intraexaminer or/and interexaminer?; Item 8. Were there blinding measurements?; Item 9. Does the study give details of methods of assessment (measurements) for each variable of interest?; Item 10. Was there a complete and adequate reporting of results, with self-explanatory tables and figures?; Item 11. Was there a statistical analysis appropriate for data?; Item 12. Was the P-value stated or confidence intervals provided?

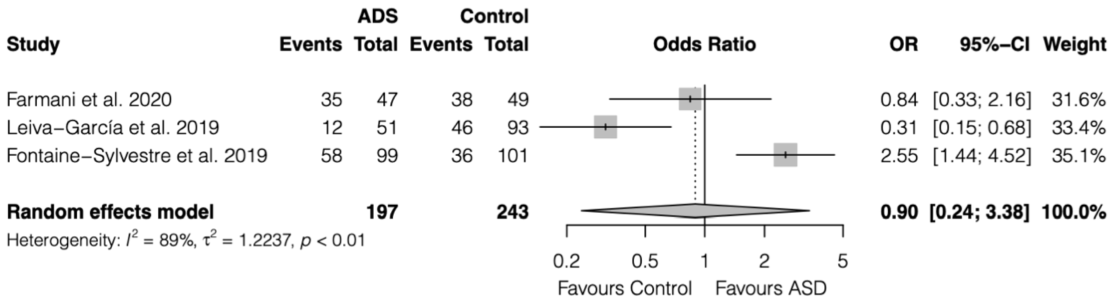

Figure S1. Forest plots for Odds Ratio of malocclusion of ASD versus control group.

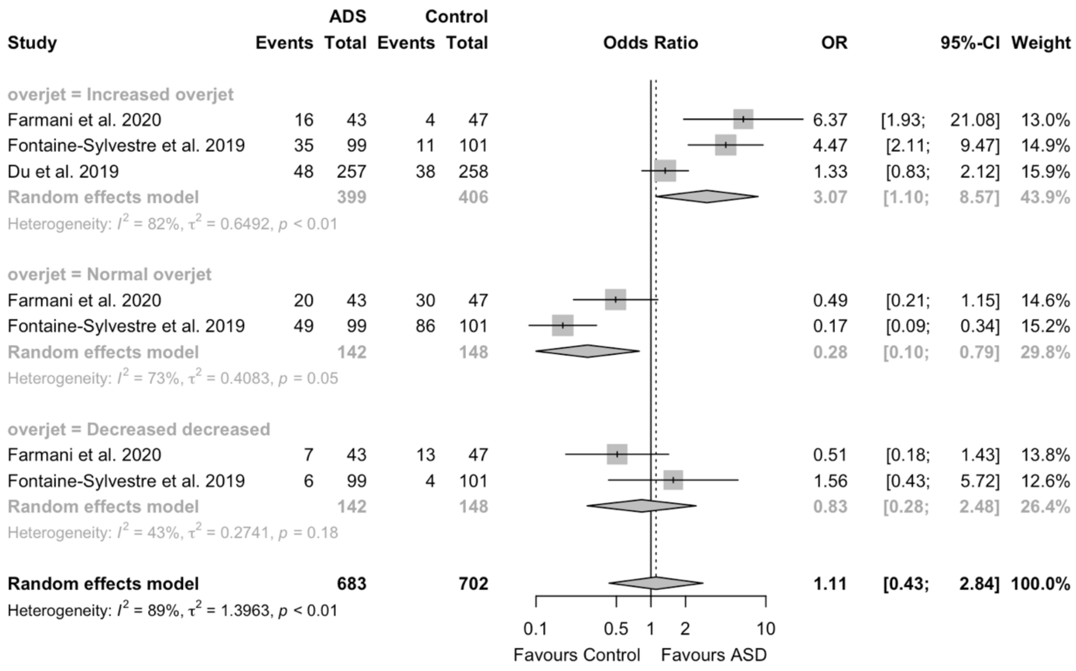

Figure S2. Forest plots for Odds Ratio of overjet of ASD versus control group. Subgroup analysis according to the increased, normal and decreased overjet.

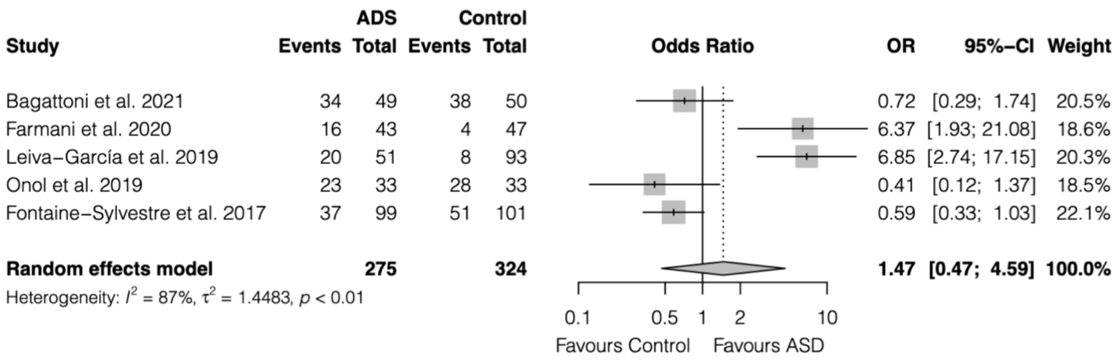

Figure S3. Forest plots for Odds Ratio of Class I of ASD versus control group.

Study	ADS Events	ADS Total	Control Events	Control Total	OR	95%-CI	Weight
Bagattoni et al. 2021	13	49	9	50	1.65	[0.63; 4.30]	20.8%
Farmani et al. 2020	16	43	4	47	6.37	[1.93; 21.08]	16.1%
Leiva−García et al. 2019	5	51	12	93	0.73	[0.24; 2.21]	17.8%
Onol et al. 2019	8	33	4	33	2.32	[0.62; 8.63]	14.2%
Fontaine−Sylvestre et al. 2019	37	99	30	101	1.41	[0.78; 2.55]	31.0%
**Random effects model**		**275**		**324**	**1.78**	**[0.97; 3.24]**	**100.0%**

Heterogeneity: $I^2 = 47\%$, $\tau^2 = 0.2137$, $p = 0.11$

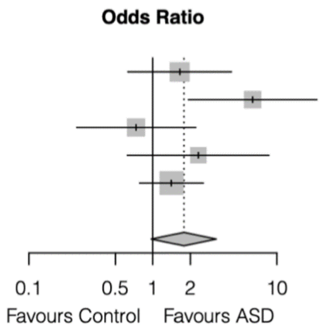

Figure S4. Forest plots for Odds Ratio of Class II of ASD versus control group.

Study	ADS Events	ADS Total	Control Events	Control Total	OR	95%-CI	Weight
Bagattoni et al. 2021	2	49	3	50	0.67	[0.11; 4.17]	9.2%
Farmani et al. 2020	7	43	13	47	0.51	[0.18; 1.43]	29.0%
Leiva−García et al. 2019	3	51	8	93	0.66	[0.17; 2.62]	16.4%
Onol et al. 2019	2	33	1	33	2.06	[0.18; 23.94]	5.1%
Fontaine−Sylvestre et al. 2019	13	99	10	101	1.38	[0.57; 3.30]	40.3%
**Random effects model**		**275**		**324**	**0.87**	**[0.50; 1.52]**	**100.0%**

Heterogeneity: $I^2 = 0\%$, $\tau^2 = 0$, $p = 0.59$

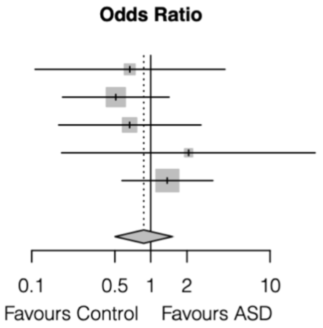

Figure S5. Forest plots for Odds Ratio of Class III of ASD versus control group.

Study	ADS Events	ADS Total	Control Events	Control Total	OR	95%-CI	Weight
**Crossbite = Anterior**							
Bagattoni et al. 2021	12	64	3	64	4.69	[1.26; 17.53]	11.1%
Fontaine−Sylvestre et al. 2017	8	99	6	101	1.39	[0.46; 4.17]	13.9%
Du et al. 2015	36	257	28	258	1.34	[0.79; 2.27]	24.8%
**Random effects model**		**420**		**423**	**1.72**	**[0.90; 3.28]**	**49.7%**
Heterogeneity: $I^2 = 34\%$, $\tau^2 = 0.1232$, $p = 0.22$							
**Crossbite = Posterior**							
Bagattoni et al. 2021	9	49	10	50	0.90	[0.33; 2.45]	15.3%
Fontaine−Sylvestre et al. 2017	13	99	5	101	2.90	[0.99; 8.48]	14.2%
Du et al. 2015	0	257	1	258	0.33	[0.01; 8.22]	2.6%
**Random effects model**		**405**		**409**	**1.38**	**[0.50; 3.81]**	**32.2%**
Heterogeneity: $I^2 = 39\%$, $\tau^2 = 0.3142$, $p = 0.19$							
**Crossbite = Anterior + Posterior**							
Farmani et al. 2020	3	47	7	49	0.41	[0.10; 1.69]	10.0%
Leiva−García et al. 2019	1	51	8	93	0.21	[0.03; 1.75]	5.5%
Onol et al. 2019	0	33	1	33	0.32	[0.01; 8.23]	2.6%
**Random effects model**		**131**		**175**	**0.33**	**[0.11; 1.00]**	**18.1%**
Heterogeneity: $I^2 = 0\%$, $\tau^2 = 0$, $p = 0.88$							
**Random effects model**		**956**		**1007**	**1.21**	**[0.71; 2.08]**	**100.0%**

Heterogeneity: $I^2 = 39\%$, $\tau^2 = 0.2335$, $p = 0.11$

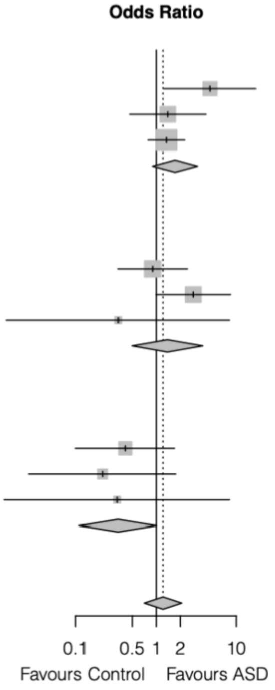

Figure S6. Forest plots for Odds Ratio of crossbite of ASD versus control group. Subgroup analysis according to the anterior, posterior, and anterior + posterior crossbite.

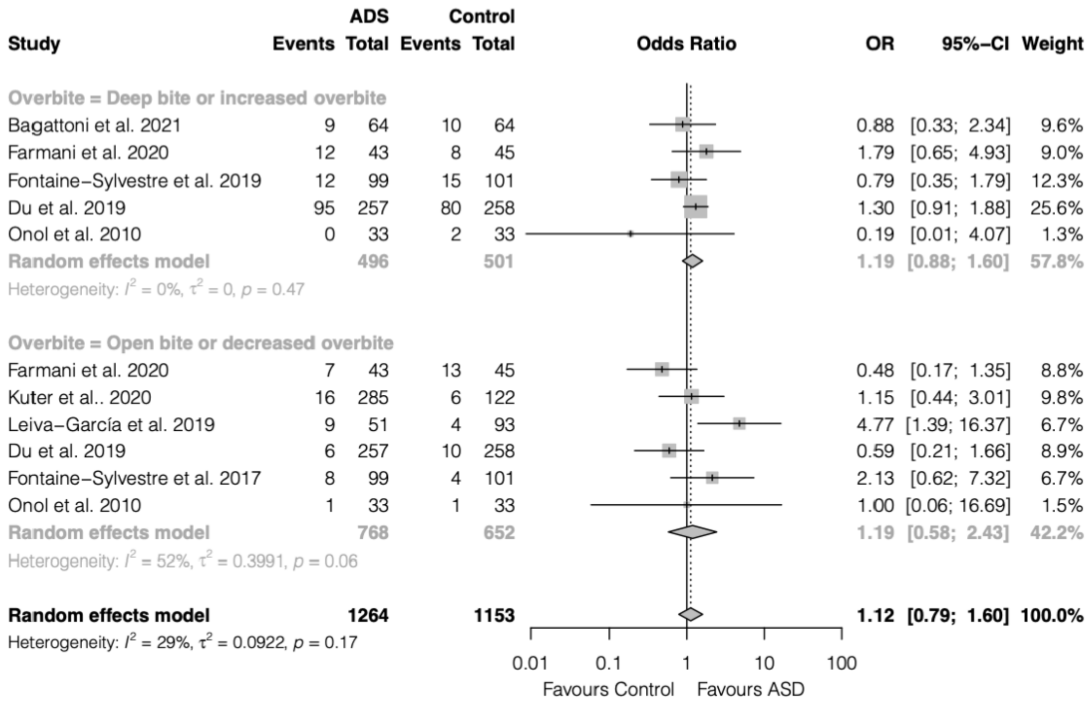

Figure S7. Forest plots for Odds Ratio of overbite of ASD versus control group. Subgroup analysis according to the deep bite or increased overbite, and open bite or decreased overbite.
